# *Saccharomyces cerevisiae* exhibiting a modified route for uptake and catabolism of glycerol forms significant amounts of ethanol from this carbon source considered as ‘non-fermentable’

**DOI:** 10.1186/s13068-019-1597-2

**Published:** 2019-10-31

**Authors:** Maximilian R. Aßkamp, Mathias Klein, Elke Nevoigt

**Affiliations:** Department of Life Sciences and Chemistry, Jacobs University Bremen gGmbH, Campus Ring 1, 28759 Bremen, Germany

**Keywords:** Glycerol, Ethanol, *S.* *cerevisiae*, Fermentation, Dihydroxyacetone pathway, Fps1/aquaglyceroporin, Redox balance, Overflow metabolism, NADH

## Abstract

**Background:**

Due to its inevitable formation during biodiesel production and its relatively high degree of reduction, glycerol is an attractive carbon source for microbial fermentation processes. However, glycerol is catabolized in a fully respiratory manner by the eukaryotic platform organism *Saccharomyces cerevisiae*. We previously engineered *S.* *cerevisiae* strains to favor fermentative metabolism of glycerol by replacing the native FAD-dependent glycerol catabolic pathway with the NAD-dependent ‘DHA pathway’. In addition, a heterologous aquaglyceroporin (Fps1 homolog) was expressed to facilitate glycerol uptake. The current study was launched to scrutinize the formation of *S. cerevisiae*’s natural fermentation product ethanol from glycerol caused by the conducted genetic modifications. This understanding is supposed to facilitate future engineering of this yeast for fermenting glycerol into valuable products more reduced than ethanol.

**Results:**

A strain solely exhibiting the glycerol catabolic pathway replacement produced ethanol at concentrations close to the detection limit. The expression of the heterologous aquaglyceroporin caused significant ethanol production (8.5 g L^−1^ from 51.5 g L^−1^ glycerol consumed) in a strain catabolizing glycerol via the DHA pathway but not in the wild-type background. A reduction of oxygen availability in the shake flask cultures further increased the ethanol titer up to 15.7 g L^−1^ (from 45 g L^−1^ glycerol consumed).

**Conclusion:**

The increased yield of cytosolic NADH caused by the glycerol catabolic pathway replacement seems to be a minimal requirement for the occurrence of alcoholic fermentation in *S.* *cerevisiae* growing in synthetic glycerol medium. The remarkable metabolic switch to ethanol formation in the DHA pathway strain with the heterologous aquaglyceroporin supports the assumption of a much stronger influx of glycerol accompanied by an increased rate of cytosolic NADH production via the DHA pathway. The fact that a reduction of oxygen supply increases ethanol production in DHA pathway strains is in line with the hypothesis that a major part of glycerol in normal shake flask cultures still enters the catabolism in a respiratory manner.

## Background

Glycerol has been considered a substrate for biotechnological processes since it is a major by-product of the transesterification process during the production of biodiesel and can be used by many microorganisms as the sole source of carbon [1]. A feature that makes glycerol particularly interesting for the fermentative production of reduced small molecules is its comparably high degree of reduction per carbon [2, 3] which generally allows higher maximum theoretical product yields in comparison to sugars [4, 5].

The yeast *Saccharomyces cerevisiae* has increasingly been employed as a production host in industrial biotechnology [6–10]. Apart from equipping this yeast with novel product formation pathways, extensive metabolic engineering incentives have been initiated in recent years to extend the organism’s range of used carbon sources with the goal to establish *S.* *cerevisiae* as a platform organism for processes based on renewable feedstocks [11–14].

Most strains of the species *S.* *cerevisiae* only seem to be able to grow on glycerol as the sole source of carbon if complex supplements such as yeast extract or amino acid mixtures are added to the medium [15–17]. In a previous study, 52 *S.* *cerevisiae* isolates were screened for their ability to utilize glycerol in synthetic medium without any of these supplements and it has been confirmed that many strains indeed do not grow at all under these conditions [18]. Still, several strains showed growth, and the strain CBS 6412 was one of the best performing strains. A haploid segregant of this strain (CBS 6412-13A) was studied in more detail. It exhibited a maximum specific growth rate (*µ*_max_) of about 0.13 h^−1^ under the conditions used [18].

**Fig. 1:**
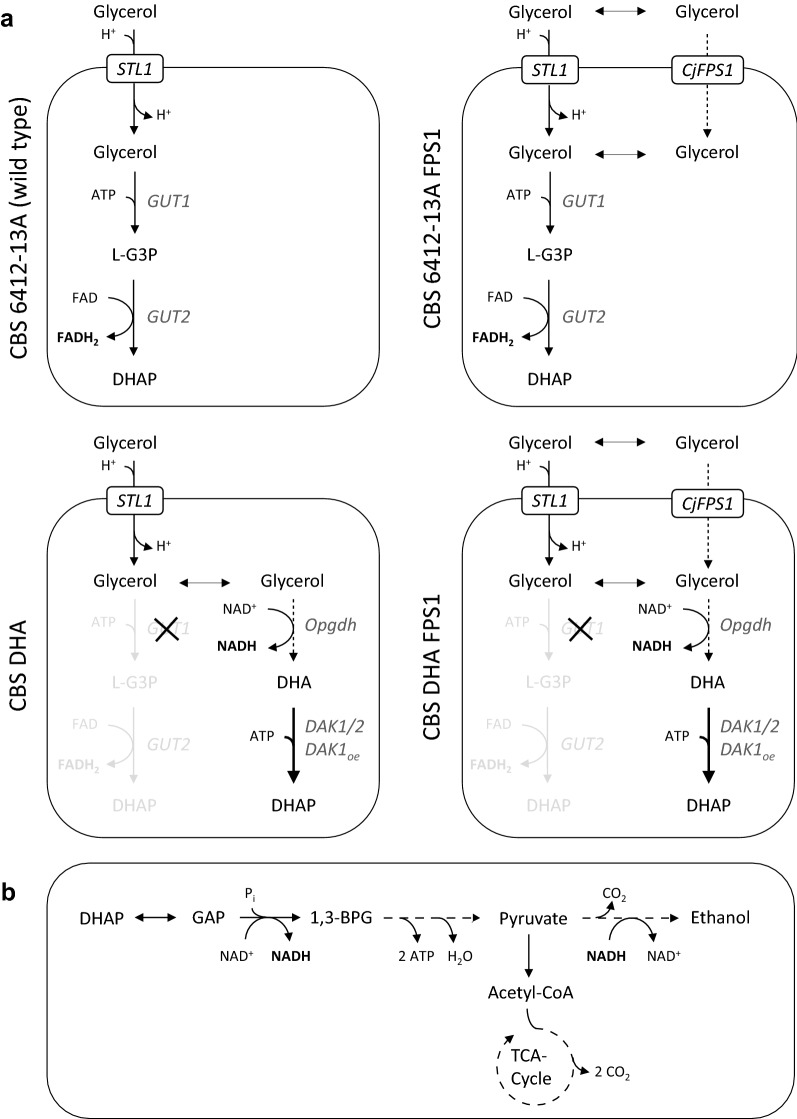
Metabolic schemes of glycerol catabolism in the used strains. **a** Glycerol catabolism (from glycerol to dihydroxyacetone phosphate) in the *S.* *cerevisiae* wild-type strain CBS 6412-13A and in engineered derivatives in which a heterologous aquaglyceroporin (CBS 6412-13A FPS1) and/or the DHA pathway was established (CBS DHA and CBS DHA FPS1). **b** Conversion of dihydroxyacetone phosphate to pyruvate (glycolysis), of pyruvate to carbon dioxide (pyruvate dehydrogenase and TCA cycle) and of pyruvate to ethanol (alcoholic fermentation). Ethanol formation has been demonstrated to only occur in strains that carry the DHA pathway as described in the main text. Dotted arrows indicate the expression of a heterologous gene and the metabolic reaction catalyzed by the respective gene product. The bold arrows indicate the overexpression of an endogenous gene (*DAK1*); and the dashed arrows represent metabolic conversions that consist of more than one enzymatic reaction. *Metabolites*: 1,3-BPG: 1,3-bisphosphoglycerate; l-G3P: l-glycerol-3-phosphate; DHA: dihydroxyacetone; DHAP: dihydroxyacetone phosphate; *Genes*: *CjFPS1*: *FPS1* homolog (aquaglyceroporin) from *C. jadinii*; *GUT1*: glycerol kinase; *GUT2*: mitochondrial (membrane bound) FAD-dependent l-G3P dehydrogenase; *DAK1*/*DAK2*: DHA kinase (*DAK1oe*: *DAK1* overexpression), *Opgdh*: glycerol dehydrogenase from *O.* *parapolymorpha*; *STL1*: glycerol/H^+^ symporter.

In *S.* *cerevisiae* wild-type cells, the uptake of glycerol is conducted by a glycerol/H^+^ symporter encoded by *STL1* [19] before it is catabolized via the l-glycerol 3-phosphate (l-G3P) pathway [18, 20] (Fig. 1a; wild-type strain CBS 6412-13A). In this pathway, a glycerol kinase (encoded by *GUT1*) and an FAD-dependent l-G3P dehydrogenase (encoded by *GUT2*) catalyze the conversion of glycerol to dihydroxyacetone phosphate (DHAP) via the intermediate l-G3P [21, 22].

It is obvious that the native l-G3P pathway of *S.* *cerevisiae* is not optimal to exploit the substrate’s attractive reducing power (for the production of reduced compounds) since part of the electrons are eventually channeled via FADH_2_ and the mitochondrial respiratory chain to oxygen. In fact, no fermentation products have ever been reported in cultivations using *S.* *cerevisiae* wild-type strains growing on glycerol. This also holds for engineered or evolved strains growing with higher *µ*_max_. For example, Ochoa-Estopier et al. [23] evolved strains for improved growth in synthetic glycerol medium (*µ*_max_ of 0.20 h^−1^) but did not detect any fermentation products even when tested under microaerobic conditions. Moreover, the CBS 6412-13A derivative, in which a heterologous aquaglyceroporin (Fps1 homolog) was expressed, achieved a *µ*_max_ of 0.18 h^−1^ but did not form any ethanol representing the natural fermentation product of *S.* *cerevisiae* [24].

To save the electrons in the form of cytosolic NADH and make them available for fermentative routes, the native glycerol catabolic pathway in *S. cerevisiae* was previously replaced by the NAD-dependent ‘DHA pathway’ [25]. The general modifications are similar to those shown in Fig. 1 for strain CBS DHA although it has to be mentioned that the actual genetic modifications leading to the depicted fluxes are slightly different in the current study. Notably, all tested *S.* *cerevisiae* strains carrying the latter modifications were able to utilize glycerol, some of them even better than their wild-type counterparts [25]. However, no ethanol formation was detected in these strains under the conditions employed (synthetic medium, ammonium sulfate as a nitrogen source, no buffering compound added, initial pH 4).

Another previously conducted study aimed at the production of 1,2-propanediol (1,2-PDO) from glycerol [26]. Here, the strain CBS 6412-13A was equipped with the DHA pathway, the heterologous aquaglyceroporin encoded by *CjFPS1* and a heterologous pathway for 1,2-PDO production. In addition, the promoter of the endogenous *TPI1* gene was replaced by a weaker one with the goal to force the metabolic flux into 1,2-PDO production. The same synthetic medium as described in Klein et al. [25] was used but an initial pH of 5 was used and 100 mM potassium hydrogen phthalate was added as a buffer to reduce medium acidification and thus to achieve higher glycerol consumption and biomass production in the shake flask cultures. Surprisingly, apart from 3.6 g L^−1^ 1,2-PDO, the constructed strain produced a considerable amount of ethanol (~ 8 g L^−1^) [26]. However, the experimental data did not allow a precise conclusion  which genetic modifications were crucial for the metabolic switch towards ethanol formation.

The initial goal of the current study was to scrutinize whether the mere replacement of the l-G3P by the DHA pathway can cause a partial fermentative metabolism of glycerol in shake flasks as soon as buffered synthetic medium is used. Moreover, the effect of an increased glycerol influx into a cell (by the expression of *CjFPS1*) was separately tested in the current study. Finally, we analyzed the effect of oxygen limitation on ethanol formation from glycerol since this reduces the re-oxidation of cytosolic NADH via respiratory routes. The generated knowledge was supposed to facilitate a better understanding of the metabolism of glycerol in DHA pathway strains and the prerequisites for glycerol fermentation i.e. ethanol formation. Although the production of ethanol from glycerol does not seem to be an attractive commercial process (particularly since the pathway is not redox neutral), the gained knowledge obtained with the natural fermentation product ethanol will guide future studies towards the production of valuable fermentation products from glycerol that are more reduced than ethanol.

## Results

### Increased cytosolic NADH generation by the DHA pathway was a minimal requirement for ethanol production from glycerol

To re-evaluate whether the glycerol catabolic pathway replacement (using the NADH-generating DHA pathway instead of the native l-G3P pathway) is a minimum requirement for alcoholic fermentation on glycerol, the strain CBS DHA was used. The latter strain (DHA pathway strain) is an engineered derivative of the wild-type strain CBS 6412-13A and was recently constructed by Aßkamp et al. [27]. In this strain, the glycerol catabolic pathway replacement was achieved by the genomic integration of cassettes for the expression of a glycerol dehydrogenase from *Ogataea parapolymorpha* (OpGDH) and the overexpression of endogenous dihydroxyacetone kinase (Dak1) as well as deletion of *GUT1* (to abolish the l-G3P pathway) [25, 27].

The strain was characterized in buffered synthetic glycerol medium (SMG_buff_). By the addition of a buffering component (100 mM potassium hydrogen phthalate) to the SMG medium and adjusting an initial pH of 5, it was previously shown that the cells in the shake flask cultivations reached significantly higher densities [26]. The use of buffered medium was able to slow down the medium acidification which can be observed when *S.* *cerevisiae* grows with ammonium sulfate as the nitrogen source. Without buffering, the pH value in batch cultivations rapidly decreases to values below 2.6 accompanied by rather low final optical densities (< 10).

**Fig. 2:**
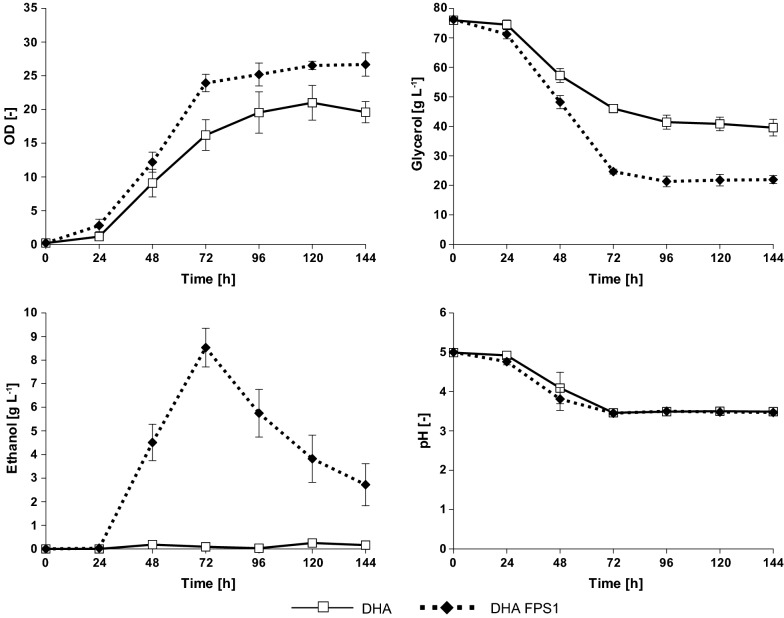
Fermentation performance of the *S.* *cerevisiae* strain CBS 6412-13A catabolizing glycerol via the ‘DHA pathway’ (DHA) and in an isogenic strain expressing *C*. *jadinii FPS1* (DHA FPS1). The shake flask experiments were conducted in synthetic media (SMG_buff_). Cells were grown in 500-mL Erlenmeyer flasks with 50 mL filling volume. Glycerol (6% v/v) was used as the sole carbon source. Mean values and standard deviations from three independent experiments are shown

As visible in Fig. 2, the strain CBS DHA produced virtually no ethanol even though buffered medium was used. However, it must be emphasized that the HPLC chromatogram always showed a small peak for ethanol which was in contrast to cultivations in unbuffered SMG (Additional file 1). It can be concluded that glycerol metabolism via the DHA pathway can induce some ethanol production but this observation cannot explain the high ethanol titers observed in the study of Islam et al. [26].

### Enhanced glycerol uptake significantly accelerated the overflow metabolism

The impact of an enhanced glycerol uptake (due to the presence of *CjFPS1*) on ethanol formation from glycerol in the DHA pathway strain was tested. Therefore, the respective expression cassette was integrated in the genome of the strain CBS DHA generating strain CBS DHA FPS1 (see “Materials and methods”). Compared to the strain CBS DHA, a remarkable increase in ethanol production was detected in strain CBS DHA FPS1. The maximum ethanol concentration of ~ 8.5 g L^−1^ was detected after 72 h of cultivation (Fig. 2). Similar results were obtained when the strain was cultivated in synthetic glycerol medium containing urea as the nitrogen source (data not shown).

The high ethanol concentration obtained for strain CBS DHA FPS1 encouraged us to also test a CBS 6412-13A wild-type derivative solely carrying the *CjFPS1* expression cassette in SMG_buff_. However, no ethanol was detected in supernatants of the respective cultivations. Taken together, the results suggest that the DHA pathway is an essential/minimal requirement for the observed overflow metabolism with the ‘non-fermentable’ carbon source glycerol but an increased glycerol influx capacity can significantly accelerate this phenomenon. The strain CBS DHA FPS1 showed a shorter lag phase compared to CBS DHA, reached a higher total biomass accumulation, consumed glycerol at a higher volumetric rate and consumed around 20 g L^−1^ more glycerol than strain CBS DHA (Fig. 2).

Notably, the ethanol titer measured in the cultivations with strain CBS DHA FPS1 decreased after reaching a maximum. This phenomenon has also previously been described by Islam et al. [26] in the strains engineered for 1,2-PDO production. The observed decrease in ethanol concentration in the current study seems to be predominantly caused by evaporation of ethanol from the medium (Additional file 2), although a certain degree of ethanol utilization cannot be excluded.

### Oxygen limitation strongly increases ethanol formation from glycerol in strain CBS DHA FPS1

**Fig. 3:**
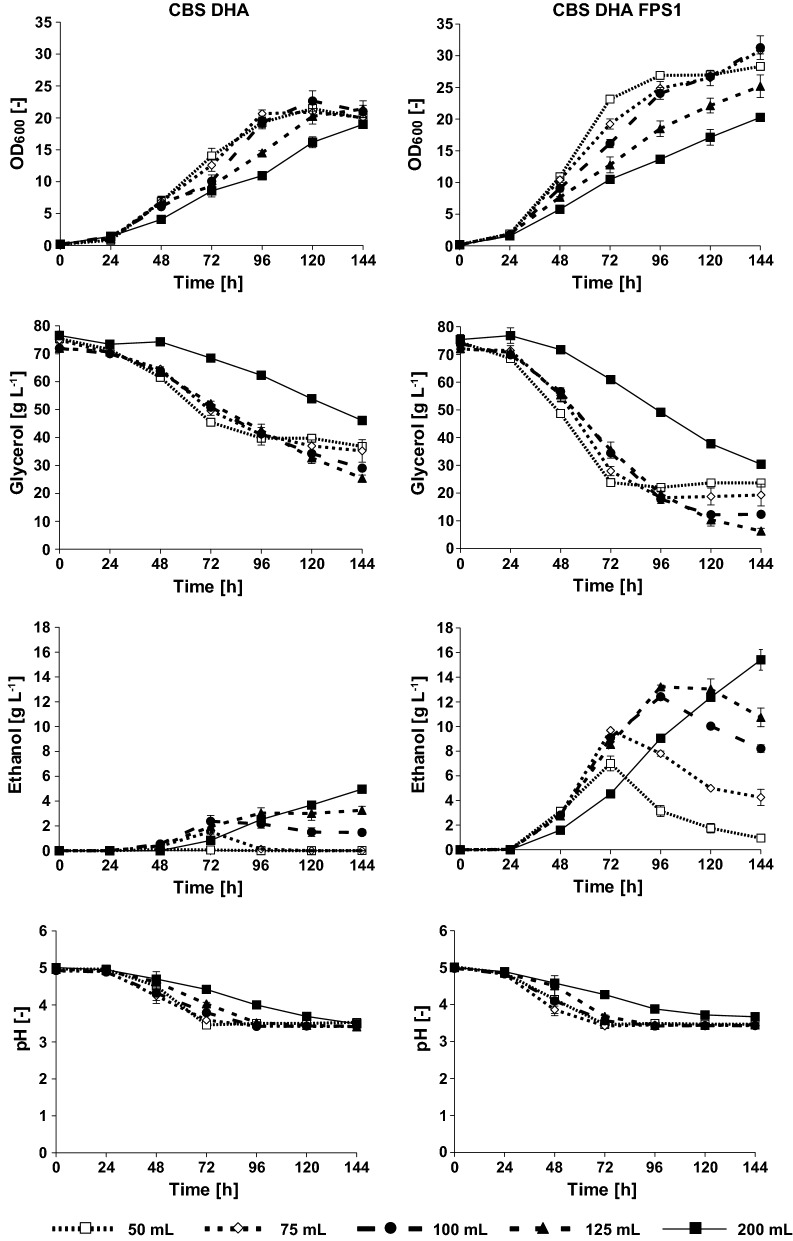
Effect of increased culture volumes (limited oxygen availability) on ethanol formation in the *S.* *cerevisiae* strains CBS DHA and CBS DHA FPS1. Experiments were conducted with 50 mL, 75 mL, 100 mL, 125 mL and 200 mL filling volume in 500-mL Erlenmeyer shake flasks in synthetic medium  where glycerol (6% v/v) was used as sole carbon source (SMG_buff_). Mean values and standard deviations from three independent experiments are shown

The fermentation of glycerol into ethanol described in the previous paragraphs relates to shake flask experiments, i.e., under aerobic conditions. Still, the increasing cell densities during the course of fermentation might have caused a constant reduction of  dissolved oxygen resulting in ethanol formation due to limitation of respiration. Thus, the question arose whether an additional reduction of the maximum oxygen transfer rate (OTR_max_) would alter the cells’ metabolism even further towards fermentation, i.e., even higher ethanol formation. A straightforward possibility to reduce OTR_max_ in shake flasks is the reduction of the surface area to volume ratio of the medium [28]. Thus, the volume of SMG_buff_ used for cultivation in the 500-mL shake flasks was stepwise increased. Both strains (CBS DHA and CBS DHA FPS1) were tested in 50 mL, 75 mL, 100 mL, 125 mL and 200 mL of cultivation medium (Fig. 3).

The results show that reducing the oxygen availability indeed increased the maximum ethanol titer (Fig. 3). Interestingly, the reduced oxygen availability also resulted in significant ethanol production in the strain CBS DHA which only formed trace amounts of ethanol when a filling volume of 50 mL was used. The highest ethanol titer for each strain was detected with the highest filling volume (200 mL) and reached a value of 5.0 ± 0.1 g L^−1^ for strain CBS DHA and 15.7 ± 0.1 g L^−1^ for strain CBS DHA FPS1. The highest ethanol yield (0.344 g g^−1^) was obtained after 96-h cultivation for strain CBS DHA FPS1.

The limited oxygen availability had a strong impact on the growth of both tested strains. The rate of biomass formation clearly decreased with increasing filling volume (Fig. 3). It was, therefore, interesting to recognize that the initial volumetric glycerol consumption and ethanol production rates were relatively independent of oxygen availability for each of the strains. The only exception was the 200 mL culture. Here, both glycerol consumption and ethanol production rates were significantly lower compared to the other tested volumes for both strains.

Another interesting finding was that the consumption of glycerol in the 50 mL culture stopped at a higher remaining substrate concentration compared to the higher volumes (75–125 mL). In the latter three cultures, the total amount of consumed glycerol increased with the culture volume. It seems that the reduced access to oxygen provides an advantage for the fitness of the cells. Future experiments will have to show whether this could be connected to the reduced production of reactive oxygen species potentially in combination with other types of stress (such as low pH) during the later phase of the cultivation.

## Discussion

As described in “Background”, it has been generally accepted that *S.* *cerevisiae* catabolizes glycerol solely in a respiratory manner [29, 30]. This seems to hold for wild-type strains and derivatives thereof, which catabolize glycerol via the l-G3P pathway. The current study demonstrates that the extra cytosolic NADH (per glycerol consumed) generated via the synthetic DHA pathway is indeed crucial for a partial shift to fermentative metabolism. However, a remarkable production of ethanol in a similarly high range as previously observed by Islam et al. [26] could not be obtained without expressing the *FPS1* gene from *C.* *jadinii* in parallel to the glycerol catabolic pathway replacement. The *CjFPS1* expression is assumed to have significantly increased the rate of glycerol uptake and cytosolic NADH generation in the DHA pathway strain thereby strongly increasing the need for the observed overflow metabolism.

The occurrence of alcoholic fermentation during aerobic growth in glucose-containing medium has been intensively studied in *S.* *cerevisiae* and is generally referred to as the “Crabtree effect” [31]. So far, this effect has been reported to occur when cells from a glucose-limited chemostat cultivation were subjected to a short glucose pulse (short-term Crabtree effect) or when cells grow in batch cultivation in excess glucose (long-term Crabtree effect) [32]. Studying the molecular basis for the regulation occurring during the Crabtree effect is challenging since it cannot be easily decoupled from the presence of glucose. Therefore, it is difficult to dissect between direct regulatory effects of glucose and indirect regulatory mechanisms triggered, e.g., by changes in the level of NADH or other intracellular metabolites. Moreover, glucose present at a concentration exceeding a certain threshold level is well known to cause huge changes in global gene expression (at the level of transcription), a phenomenon that is also known as glucose repression [33, 34]. With regard to the long-term Crabtree effect, the contributions of changed protein concentrations caused by the glucose repression and of metabolic regulation of enzymes caused by changes in metabolite levels are highly interwoven. The fact that ethanol production can now be observed even in the absence of glucose (i.e., when cells catabolize glycerol via the DHA pathway) provides a promising future opportunity to further dissect the molecular reasons of the Crabtree effect in *S.* *cerevisiae*.

One theory for explaining the Crabtree effect is applicable even in the absence of glucose repression. This theory assumes that an increased rate of glycolysis together with a limitation in respiratory capacity leads to an imbalanced NADH/NAD^+^ ratio and pyruvate accumulation [32, 35–38]. The quick response during the short-term Crabtree effect (also known as metabolic overflow) seems to be regulated by a rapid change of enzymatic activities in response to NADH overflow. When a *S.* *cerevisiae* strain grows on glycerol and, most importantly, uses the DHA pathway, the higher yield of cytosolic NADH generated per carbon consumed (together with its accelerated formation due to increased glycerol influx by CjFps1 activity) is supposed to be significantly increased compared to a strain using the l-G3P pathway and to cause significant ethanol formation from the ‘non-fermentable’ carbon source glycerol. This effect might have been triggered by the same ‘NADH overflow’ accompanied by the respective regulatory processes as described during the short-term Crabtree effect on glucose. In this context, it is interesting to note that Hagman and Piškur [37] suggest overflow metabolism to be the fundamental mechanism behind the long- and short-term Crabtree effects and to be evolutionary much older than glucose repression of respiration. None of the strains that use the native l-G3P pathway for glycerol catabolism was capable to produce ethanol from glycerol (even with additional expression of an *FPS1* homolog and cultivation under oxygen-limited conditions). These results seem to confirm that the NADH-generating glycerol oxidation step was indeed crucial for the switch to alcoholic fermentation when DHA pathway strains grow on glycerol.

In the current study, it has also been shown that lower oxygen transfer rates resulted in a further increase in ethanol formation in both strains CBS DHA and CBS DHA FPS1. This is in line with the fact that the capacity of any respiratory mechanism for re-oxidizing cytosolic NADH such as the external NADH dehydrogenases or the l-G3P shuttle must become limiting as soon as the availability of the final electron acceptor is strongly decreased. Under oxygen-limited conditions, re-oxidation of cytosolic NADH via fermentation is the only possible route to maintain redox balance and to sustain carbon flux through glycolysis. Otherwise, the cells have to respond with a reduction in growth rate. Interestingly, the latter has been observed in an *nde1*∆ deletion mutant of strain CBS DHA supposed to have reduced activity of the external NADH dehydrogenase when it was cultivated in synthetic glycerol medium [27]. This mutant did not produce any ethanol even if buffered medium was used (data not shown). It is still not clear, why the strain CBS DHA can (at least partly) switch to alcoholic fermentation when oxygen availability is strongly reduced but not if a major route for respiratory re-oxidation of cytosolic NADH is abolished by deleting *NDE1*. It might be that the abolishment of Nde1 activity is too severe or causes pleiotropic effects when the mutant grows in glycerol medium.

Ethanol production from glycerol in engineered strains of *S.* *cerevisiae* has already been reported by Yu et al. [39]. The genetic modifications considered causative for ethanol formation were the overexpression of *GCY1* in combination with the overexpression of the endogenous dihydroxyacetone kinase. As *GCY1* is assumed to encode an NADP-dependent glyceraldehyde reductase [40–42], the results of Yu et al. [39] are counterintuitive. Moreover, these authors used a medium containing amino acids and nucleic bases. The presence of these supplements has previously been shown to significantly affect glycerol utilization by wild-type *S.* *cerevisiae* strains [15, 18] and might thus have also affected the production of ethanol.

The current study demonstrated for the first time alcoholic fermentation of glycerol by *S.* *cerevisiae* in pure synthetic medium. The highest titer obtained here was ~ 15 g L^−1^ ethanol by growing the strain CBS DHA FPS under oxygen-limited conditions (200 mL of culture medium). This corresponds to an ethanol yield of 0.34 g/g glycerol. Exclusively considering the carbon, 45.7% can be found in ethanol and 37.5% in biomass at this point in time. Future studies in bioreactors will focus on quantifying carbon dioxide formation to allow the calculation of a solid carbon balance. We are currently working on establishing a redox neutral pathway which allows glycerol fermentation without the requirement of oxygen.

## Conclusions

Genetic modifications leading to an increased yield and production rate of cytosolic NADH induce a metabolic overflow similar to the Crabtree effect in *S.* *cerevisiae* even if the cells grow on glycerol, a carbon source considered as non-fermentable for this species so far. The replacement of the l-G3P pathway by the DHA pathway for glycerol utilization seems to be a minimal requirement for ethanol formation in synthetic glycerol medium. The obtained results might provide interesting future opportunities to study the Crabtree effect uncoupled from glucose repression and for the valorization of glycerol-containing feedstocks.

## Materials and methods

### Strains, medium composition and general cultivation conditions

All *S.* *cerevisiae* cells (Table 1) were routinely grown on solid (15 g L^−1^ agar) or in liquid YPD medium containing 10 g L^−1^ yeast extract, 20 g L^−1^ peptone, and 20 g L^−1^ glucose. Liquid cultures were incubated in an orbital shaker at 200 rpm and 30 °C. Plasmid carrying *E.* *coli* DH5α strains were grown at 250 rpm and 37 °C in lysogeny broth (LB) (10 g L^−1^ peptone, 5 g L^−1^ yeast extract and 10 g L^−1^ NaCl, pH 7.0) containing 100 mg L^−1^ ampicillin for selection purposes.

**Table 1:** List of all *S.* *cerevisiae* strains and all plasmids used in this study

Strain	Genome modifications	References
CBS 6412-13A	–	Swinnen et al. [18]
CBS 6412-13A FPS1	*YGLCτ3:: PGK1p*-*CjFPS1*-*RPL15At:loxP*-*ble*-*loxP*	This study
CBS DHA	*YPRC*Δ*15::ACT1p*-*DAK1*-*TPS1t*; *YPRCτ3::TEF1p*-*Opgdh*-*CYC1t; gut1::loxP*	Aßkamp et al. [27]
CBS DHA FPS1	*YPRC*Δ*15::ACT1p*-*DAK1*-*TPS1t*; *YPRCτ3::TEF1p*-*Opgdh*-*CYC1t; gut1::loxP; YGLCτ3::PGK1p*-*CjFPS1*-*RPL15At:loxP*-*ble*-*loxP*	This study

Plasmid	Relevant characteristics	References
p426-SNR52p-gRNA-YGLCτ3-SUP4t-hphMX	gRNA targeting *YGLCτ3; hygromycin B* resistance	Klein et al. [24]
pUC18-PGK1p-*CjFPS1*	*CjFPS1* under control of *PGK1p* and *RPL15At*	Islam et al. [26]
p414-TEF1p-Cas9-CYC1t-nat1	Cas9 from *S. pyogenes*; nourseothricin resistance	Klein et al. [24]

### Media for analyzing yeast growth and fermentation behavior with glycerol as the sole carbon source

The synthetic media were based on the medium described by Verduyn et al. [43] containing: 5 g L^−1^ (NH_4_)_2_SO_4_, 3 g L^−1^ KH_2_PO_4_, 0.5 g L^−1^ MgSO_4_·7H_2_O, 15 mg L^−1^ EDTA, 4.5 mg L^−1^ ZnSO_4_·7H_2_O, 0.84 mg L^−1^ MnCl_2_·2H_2_O, 0.3 mg L^−1^ CoCl_2_·6H_2_O, 0.3 mg L^−1^ CuSO_4_·5H_2_O, 0.4 mg L^−1^ NaMoO_4_·2H_2_O, 4.5 mg L^−1^ CaCl_2_·2H_2_O, 3 mg L^−1^ FeSO_4_·7H_2_O, 1 mg L^−1^ H_3_BO_3_, and 0.1 mg L^−1^ KI. As carbon source, 60 mL L^−1^ glycerol was added to the medium. To buffer the medium, 20.5 g L^−1^ potassium hydrogen phthalate (C_8_H_5_KO_4_) was added (SMG_buff_). After autoclaving of this basal medium, a filter-sterilized vitamin solution was added. The final concentrations of vitamins in the medium were 0.05 mg L^−1^ d-(+)-biotin, 1 mg L^−1^ d-pantothenic acid hemicalcium salt, 1 mg L^−1^ nicotinic acid, 25 mg L^−1^ myo-inositol, 1 mg L^−1^ thiamine chloride hydrochloride, 1 mg L^−1^ pyridoxine hydrochloride, and 0.2 mg L^−1^ 4-aminobenzoic acid. The pH was adjusted to 5.0 using 2 M H_3_PO_4_.

### Construction of strain CBS DHA FPS1

Plasmids (Table 1) were isolated from *E.* *coli* overnight cultures using the GeneJET™ Plasmid Miniprep Kit (Thermo Fisher Scientific, Waltham, Massachusetts, USA). The *CjFPS1* expression cassette was amplified from the plasmid pUC18-*PGK1p*-*CjFPS1* [26] with Phusion^®^ High-Fidelity DNA Polymerase (NEB, Frankfurt am Main, Germany) according to the manufacturer’s protocol. The GeneJET™ PCR Purification Kit (Thermo Fisher Scientific) was afterwards used to purify the PCR product. Transformation of *S.* *cerevisiae* was performed using the lithium acetate method [44]. The expression cassette was integrated in the genome of strain CBS DHA [27] at position *YGLCτ3* on chromosome VII using the CRISPR/Cas9 method described by DiCarlo et al. [45] and adapted by Klein et al. [25]. First, the strain was transformed with p414-TEF1p-Cas9-CYC1t-nat1 for expression of Cas9. In a second step, the resulting strain was transformed with the p426-SNR52p-gRNA.YGLCτ3-SUP4t-hphMX [26] for gRNA expression and the PCR-amplified expression cassette for *CjFPS1*. The latter contained PCR-generated regions upstream of the used *PGK1* promoter and downstream of the *RPL15A* terminator that were homologous to sequences on either side of the introduced double-strand break at the target site for integration by homologous recombination. Correct integration of the cassette was verified by diagnostic PCR. Single colonies obtained after transformation were re-streaked. Cells from these plates were used for the isolation of genomic DNA using a protocol adapted from Hoffman and Winston [46] and described in Swinnen et al. [18] in more detail. Diagnostic PCRs were performed using One*Taq* Quick-Load DNA Polymerase (NEB). The primers (Additional file 3) bind upstream and downstream of the targeted genomic integration site and within the integrated *CjFPS1* expression cassette, respectively.

### Characterization of *S.* *cerevisiae* in shake flask batch cultivation

Cells from single colonies were transferred to 5 mL of SMG_buff_ in a 10-mL glass tube and cultures incubated by shaking at 200 rpm and 30 °C for 48 h. From this pre-culture, a 15 mL intermediate culture using the same medium in a 100-mL Erlenmeyer flask was inoculated adjusting an OD_600_ of 0.2. This culture was grown under the same conditions for another 48 h. For experiments aiming at limiting the oxygen transfer by increased filling volumes, two 15 mL intermediate cultures in 100-mL Erlenmeyer flasks were inoculated from the same pre-culture and combined before preparing the respective main culture. For main culture preparation, an appropriate culture volume (to adjust to an OD_600_ of 0.2 in the main culture) was centrifuged at 800 *g* for 5 min and the supernatant was discarded. The cell pellet was then resuspended in fresh SMG_buff_ in a 500-mL Erlenmeyer flask. The final cultures had a volume of 50 mL, while additional filling volumes of 75 mL, 100 mL, 125 mL and 200 mL culture medium were used for the experiment analyzing the influence of oxygen limitation. Samples of 1.2 mL were taken every 24 h for OD_600_ and pH determination as well as for HPLC analysis.

### Quantification of ethanol and glycerol via HPLC

The samples (1.2 mL culture supernatant) were filtered through 0.2-μm Minisart RC membrane filters (Sartorius, Göttingen, Germany) and stored at − 20 °C until analysis. Detection and quantification of glycerol and ethanol were performed using a Waters HPLC system (Eschborn, Germany) consisting of a binary pump (Waters 1525), an injector system (Waters 2707) and a Waters column heater module WAT038040 and a refractive index detector (Waters 2414). An Aminex HPX-87H cation-exchange column (Biorad, München, Germany) coupled to a Micro-guard^®^ cation-exchange column (Biorad) was used for chromatography. As a solvent, 5 mM H_2_SO_4_ at a flow rate of 0.6 mL min^−1^ was used. The column was kept at 45 °C. A sample volume of 20 μL was injected. Under these conditions, the retention times were about 13.5 min for glycerol and 22.7 min for ethanol. Data were analyzed using the Breeze 2 software (Waters).

## Supplementary information

**Additional file 1.** Growth, glycerol consumption and ethanol formation of a wild-type CBS 6412-13A strain with additional expression of an aquaglyceroporin from *C.* *jadinii* (*CjFPS1*). Cells were grown in 50 mL SMG_buff_ in a 500 mL shake flask. Glycerol (6% v/v) was used as the sole carbon source. Mean values and standard deviations from three independent biological replicates are shown.

**Additional file 2.** Comparison of ethanol evaporation in SMG_buff_ with ethanol decline in cultivations of the *S.* *cerevisiae* strain CBS DHA FPS1 in the same medium. For recording the evaporation of ethanol 35 g L^−1^ were dissolved in 50 mL medium and incubated in 500 mL shake flasks under the same cultivation conditions as used for the fermentations with the *S. cerevisiae* strains (see “Materials and methods”).

**Additional file 3.** List of all primers used in this study.

## Data Availability

The datasets used and/or analyzed during the current study are available from the corresponding author upon reasonable request.

## Abbreviations

DHA: dihydroxyacetone
DHAP: dihydroxyacetone phosphate
HPLC: high-pressure liquid chromatography
l-G3P: l-glycerol 3-phosphate
LB: lysogeny broth
OD_600_: optical density determined at a wavelength of 600 nm
OTR_max_: maximum oxygen transfer rate
PCR: polymerase chain reaction
*μ*_max_: maximum specific growth rate
SMG: synthetic glycerol medium
SMG_buff_: SMG plus 100 mM potassium hydrogen phthalate
YPD: yeast extract peptone dextrose
