# Supplementary material for: Saccharomyces cerevisiae exhibiting a modified route for uptake and catabolism of glycerol forms significant amounts of ethanol from this carbon source considered as ‘non-fermentable’
Source: Biotechnol Biofuels. 2019 Oct 31;12:257. doi: 10.1186/s13068-019-1597-2 (PMC6822349; doi:10.1186/s13068-019-1597-2)
Supplement: Supplementary file 1 — Additional file 1. Growth, glycerol consumption and ethanol formation of a wild-type CBS 6412-13A strain with additional expression of an aquaglyceroporin from C. jadinii (CjFPS1). Cells were grown in 50 mL SMGbuff in a 500 mL shake flask. Glycerol (6% v/v) was used as the sole carbon source. Mean values and standard deviations from three independent biological replicates are shown. [file 13068_2019_1597_MOESM1_ESM.docx]

**Additional file 1**

**Growth, glycerol consumption and ethanol formation of a wild-type CBS 6412‑13A strain with additional expression of an aquaglyceroporin from *C. jadinii* (*CjFPS1*).** Cells were grown in 50 mL SMG_buff_ in a 500 mL shake flask. Glycerol (6% v/v) was used as sole carbon source. Mean values and standard deviations from three independent replicates are shown.
