# Supplementary material for: Saccharomyces cerevisiae exhibiting a modified route for uptake and catabolism of glycerol forms significant amounts of ethanol from this carbon source considered as ‘non-fermentable’
Source: Biotechnol Biofuels. 2019 Oct 31;12:257. doi: 10.1186/s13068-019-1597-2 (PMC6822349; doi:10.1186/s13068-019-1597-2)
Supplement: Supplementary file 2 — Additional file 2. Comparison of ethanol evaporation in SMGbuff with ethanol decline in cultivations of the S. cerevisiae strain CBS DHA FPS1 in the same medium. For recording the evaporation of ethanol 35 g L−1 were dissolved in 50 mL medium and incubated in 500 mL shake flasks under the same cultivation conditions as used for the fermentations with the S. cerevisiae strains (see “Materials and methods”). [file 13068_2019_1597_MOESM2_ESM.docx]

**Additional file 2**

**Evaporation of ethanol in SMG_buff_ and time-course of ethanol concentration detectable in supernatants of cultivations with the *S. cerevisiae* strain CBS DHA FPS1 in the same medium.** For recording the evaporation of ethanol 35 g L^-1^ were dissolved in 50 mL medium and incubated in 500 mL shake flasks under the same cultivation conditions as used for the fermentations with the *S. cerevisiae* strains (see Material and Methods).
