# Supplementary material for: Saccharomyces cerevisiae exhibiting a modified route for uptake and catabolism of glycerol forms significant amounts of ethanol from this carbon source considered as ‘non-fermentable’
Source: Biotechnol Biofuels. 2019 Oct 31;12:257. doi: 10.1186/s13068-019-1597-2 (PMC6822349; doi:10.1186/s13068-019-1597-2)
Supplement: Supplementary file 3 — Additional file 3. List of all primers used in this study. [file 13068_2019_1597_MOESM3_ESM.docx]

**Additional file 3**

**PCR primers used in this study.** 5’ overhangs used for generating overlapping PCR products to regions in the *S. cerevisiae* genome (for *in vivo* homologous recombination) are underlined. (Primer Numbers according to the Nevoigt Lab Primer collection).

| **Primer No.** | | **Name** | **Sequence 5’ – 3’** | |
| --- | --- | --- | --- | --- |
| *Primers for the integration of the CjFPS1 expression cassettes into the YGLCτ3 locus on chromosome VII* | | | |  |
| 390 | CHRVII-PGK1p-F | | CGCGGTAAATTCTTAAGGCCATATTTCAGGTAGGAACCATCGTCAACAATTGCTACAACGGAAGTACCTTCAAAGAATGGGGTCTT |  |
| 392 | RPL15At-loxP-F | | ATTAAGGGTTGTCGACCTGCAGCGTACGAAGCTTCAGCTGGGGAAAAACGGGAAGAAAAGGAAAGA |  |
| *Primers for the verification of the CjFPS1 expression cassettes into the YGLCτ3 locus on chromosome VII* | | | |  |
| 179 | HAA1 OE check F1 | | AGCGTTCGTTCTATGCCTCT |  |
| 181 | HAA1 OE check R1 | | AAGAACCAGAATGGCAGGAC |  |
| 359 | PjFPS1-VER 2 | | GACCCTTGGTGTTCAAGTA |  |
| 708 | PjFPS1-F-qPCR | | GCTGTTCTGCGGGTATCTCT |  |
